# Molecular Imaging: *In Vivo* Agents for the Diagnosis and Treatment of Cancer

**DOI:** 10.1155/2018/8541915

**Published:** 2018-10-22

**Authors:** D. Haeusler, C. Decristoforo, J. Frost, S. Gobalakrishnan, Y. Y. Huang

**Affiliations:** ^1^WISIA-Women in Science-an Interdisciplinary Association, Vienna, Austria; ^2^Medical University of Innsbruck, Innsbruck, Austria; ^3^Johns Hopkins University, Baltimore, MD, USA; ^4^Virginia Commonwealth University, Richmond, VA, USA; ^5^National Taiwan University Hospital, Taipei, Taiwan

Molecular imaging continues to advance the goal of improving diagnosis and treatment in cancer. This special issue examines a cross section of the current basic and clinical research across imaging modalities, probes, and molecular targets. The issue articles permit comparison of the advantages and limitations of varied modalities, including PET, SPECT, CT, MRI, ultrasound imaging, and fluorescence imaging. The corresponding agents in development are able to interact with many targets of relevance to cancer. Highly sensitive and specific *in vivo* agents permit visualization of receptor systems, enzymes, and proteins involved in cancer initiation, maintenance, and spread. As another dimension of cancer targeting, molecular probes for the tumor microenvironment, including stromal, endothelial, and immune cells, are increasingly recognized as key factors in attacking cancer.

The caleidoscope of targets and methods available to achieve these goals is exemplified in this special issue. The topics cover the development of molecular imaging agents targeting the cholecystokinin 2 receptor (CCK2R), the adenosine A3 receptor (A3R), and the human epidermal growth factor receptor 2 (HER2). Cancer stroma targeting is addressed with molecular probes for the vascular cell adhesion molecule 1 (VCAM-1) and endoglin (CD105), a proangiogenic growth factor, which are both overexpressed in a variety of malignancies.

The tools used in the selection of displayed articles were molecules, small peptides, engineered proteins, intact antibodies and antibody fragments, nanoparticles, and microbubbles, demonstrating the breadth of targeting scaffolds currently to investigators and soon to cancer clinicians.

Each paper included in this special issue approaches the challenge of molecular imaging in diagnosis and treatment of cancer in a different way, focusing on new molecules, innovative labelling strategies, and characterization in disease models. Each contribution stands on its own as a marker for the next advances in cancer understanding and patient care.

The editors have endeavored to have the authors clearly point up the advantages of the current technologies and demonstrate the needs for advancement to the next stages of development. We hope this effort will stimulate the reader to think hard about their own research and how the community of cancer imaging investigators can continue progress from proof of concept to clinical use.

Finally, we would like to acknowledge the strong efforts of all the reviewers who supported this special issue through their careful, insightful, and timely reviews.

This issue was edited by a group of reasearchers across the world who shared the aim to shed more light on the omnipresent unsolved cancer problem. Apart from our own research in the field of molecular imaging and cancer, it was a pleasure to conceive, organize, and edit this special issue. Last but not least, we conclude that, in molecular imaging of cancer, highly promising tools are being evaluated; as a next step, we need to use them in clinical practice.

